# Single-use and six-week effects of a non-tobacco nicotine pouch on dental plaque characteristics: a randomized crossover study with observational follow-up

**DOI:** 10.1186/s12903-026-08119-7

**Published:** 2026-03-24

**Authors:** Sara Alizadehgharib, Anna Lehrkinder, Ali Alshabeeb, Peter Lingström

**Affiliations:** 1 Department of Oral Microbiology and Immunology, Institute of Odontology, Sahlgrenska Academy, University of Gothenburg, Gothenburg, Sweden; 2 Department of Cariology, Institute of Odontology, Sahlgrenska Academy, University of Gothenburg, Gothenburg, Sweden

**Keywords:** Acidogenicity, Caries prevention, Dental plaque, Nicotine pouch

## Abstract

**Background:**

This study measures the short- and long-term acidogenicity in dental biofilms of tobacco-based snus users who switch to nicotine pouches (ZYN^®^ Dry) for delivering nicotine. ZYN Dry contains microcrystalline cellulose (MCC) and maltitol rather than grounded tobacco leaves. Although MCC and maltitol are not typically associated with changes in biofilm acidogenicity, prolonged exposure (30–60 min) may cause vulnerability and increase the caries risk.

**Methods:**

This study consists of two parts. Part A was an open-label, randomized, four-way crossover study involving single-dose administration in 18 participants. It tested flavored and unflavored ZYN products as well as 10% sucrose and 10% xylitol rinses as controls. The pouch was placed in the vestibule for 60 min, while the control mouth rinses were performed for one minute each. Part B was an open-label, observational, and follow-up study which lasted for six weeks and included 56 participants. Exposure to 10% sucrose was tested at baseline (week 0) and after 2, 4, and 6 weeks of ZYN use. Dental plaque acidogenicity was measured on two approximal surfaces in the upper jaw at baseline and up to 60 min after administration using the microtouch method. Areas under the curve for pH 7.5 were calculated (AUC_7.5_). Six tooth surfaces were used to score the amount of plaque (0–3). Ethical approval and informed consent were obtained.

**Results:**

After single-dose administration (Part A), AUC_7.5_ for dental plaque acidogenicity was significantly less pronounced for the ZYN products and xylitol compared to sucrose. There were no statistical differences between the flavored and unflavored ZYN products. Ad libitum administration of ZYN (Part B) resulted in a significant reduction in the AUC_7.5_ for plaque acidogenicity compared to baseline. No statistically significant changes in plaque amount were observed during the six-week study.

**Conclusions:**

In healthy tobacco-based snus users, dental plaque acidogenicity decreased following ad libitum use of ZYN nicotine pouches, suggesting a reduced acidogenic response under the conditions of this study.

**Trial registration:**

This clinical trial was retrospectively registered at: https://www.isrctn.com/ (ISRCTN16087707).

## Introduction

Non-combustible nicotine-containing products, such as e-cigarettes, tobacco-based snus, and nicotine pouches, are becoming increasingly popular. Current clinical evidence suggests that habitual use of tobacco-based snus is unlikely to influence biofilm acidogenicity [1]. This lack of influence may be due to factors such as nicotine’s minimal effect on biofilm acidogenicity and the fact that tobacco does not seem to act as a substrate for oral microflora. Additionally, snus contains food-approved pH-regulating substances, such as sodium carbonate, which help to maintain a relatively high pH (about pH 8.0–8.5) in the snus pinch/pouch.

The development of caries is associated with the dental biofilm’s ability to generate weak organic acids, which lower the pH of the surrounding environment, leading to demineralization [2–5]. These factors may help explain why caries do not seem to be more common among snus users compared to non-tobacco users. In fact, a previous study by Hellqvist et al. demonstrated that the use of nicotine-containing snus products increases plaque pH [6]. Additionally, snus users generally have fewer decayed and filled tooth surfaces compared to non-tobacco users, supporting the suggestion that Swedish snus does not increase the risk of dental caries [7]. On the other hand, previous investigations have revealed that nicotine levels in the saliva of individuals who use smokeless tobacco may promote the growth and metabolic activity of *Streptococcus mutans* (*S. mutans*), increasing the risk of dental caries [8, 9].

In recent years, non-tobacco-based nicotine pouches have become an alternative to tobacco-containing snus. These products, available in parts of Europe and the United States (U.S.), use synthetically produced or tobacco-derived nicotine rather than tobacco leaves [10, 11]. In the U.S., one of the market-leading brands of non-tobacco-based pouches is ZYN^®^ [12]. These products share the same physical characteristics and pH as conventional tobacco-based snus and are used in the same way, i.e., the pouch is placed in the upper sulcus for up to 60 min. The nicotine pouches deliver nicotine at similar concentrations and speed as conventional snus [13]. However, the matrix carrying the nicotine in ZYN differs from that of conventional snus. The ZYN matrix consists of microcrystals of maltitol and microcrystalline cellulose (MCC) rather than ground tobacco leaves [14]. Since the ZYN matrix does not use tobacco leaf, it does not contain any tobacco-specific nitrosamines or polycyclic aromatic hydrocarbons [14]. Maltitol and MCC, when used in food, have not previously been associated with changes in the acidogenicity or composition of biofilms [7, 8]. ZYN also contains food-grade flavourings and sweeteners such as acesulfame K [14]. Acesulfame K can suppress the growth and biofilm formation of cariogenic bacteria and has even been suggested to be an anticaries agent [15].

While no negative effects related to dental caries have been reported concerning the various ingredients, the prolonged exposure (about 30–60 min) associated with ZYN use constitutes a different type of exposure. Although there are no initial reasons to believe that ZYN use will negatively impact biofilm acidogenicity, it is reasonable to thoroughly evaluate this potential in a controlled clinical trial, especially since ZYN is marketed as a consumer product.

This study evaluates the impact of the non-tobacco-based nicotine pouch product ZYN on biofilm acidogenicity. The study was conducted in two parts (A and B). Part A assessed dental plaque acidogenicity after short-term exposure (60 min) to the study products (a flavored and an unflavored ZYN product), 10% sucrose (positive control), and 10% xylitol (negative control). Part B assessed dental plaque acidogenicity during 6 weeks of ad libitum flavored and unflavored ZYN product use.

## Materials and methods

### Data quality assurance

The study was monitored by CTC Clinical Trial Consultants AB (CTC). The study protocol was performed in accordance with the Helsinki Declaration of Human Rights and was approved by the Regional Ethical Review Board, Gothenburg, Sweden (Dnr 778 − 17). The study protocol was retrospectively registered approximately 2–3 weeks after enrolling the first participant, during the initial phase of the study on 28 November 2017 in the ISRCTN clinical trial registry (ISRCTN16087707). Healthy adults were recruited using an advertisement in the local newspaper. Following a telephone interview to evaluate eligibility, potential participants were invited to a Screening visit where they submitted a Health Declaration. The participants received oral and written information about the study and signed an informed consent form. A screening number was automatically generated in the electronic case report form and assigned to each subject upon receipt of informed consent at the screening visit (Visit 1). This number allowed identification of subjects regardless of their eligibility status. Subjects who were enrolled and randomized were subsequently assigned a randomization number.

Before enrolling the first participant in the study, a representative from the sponsor conducted a study initiation visit at the research clinic. The research site received periodic visits from a CTC monitor, who had direct access to clinical records and original data to conduct source data verification. All forms were thoroughly reviewed to ensure the completeness of the recorded data, including addressing any missing or inconsistent entries. The study duration was 06 November 2017–05 June 2018.

### Participants

The screening population comprised 83 female and male volunteers (for Parts A and B), with a mean age of 33.0 ± 10.6 years. The inclusion criteria were: aged > 19, being in good health, having used tobacco-based snus for more than one year with weekly consumption of three or more snus cans with nicotine content < 1% or two or more cans with nicotine content > 1%), and having normal stimulated salivary secretion rate (*≥* 0.7 ml/min.). In addition, female participants were required to provide a negative pregnancy test result. The following exclusion criteria were used: history or presence of diagnosed hypertension or any cardiovascular disease; surgery within six months of the screening visit that, in the opinion of the investigator, could negatively impact the subject’s participation in the clinical study; any surgical or medical condition, which, in the judgment of the clinical investigator, might interfere with the absorption, distribution, metabolism, or excretion of nicotine; pregnant; allergy towards composite materials; and antibiotic use during or within the last four weeks before the study period.

The participants in both parts of the study were instructed to refrain from approximal tooth cleaning for the 72 hours before the visits and toothbrushing for the 48 hours before the visits. No eating or drinking was allowed two hours before the visits.

### Study design

This study consisted of two components: an interventional randomized crossover trial (Part A) and a prospective within-subject observational study (Part B). No formal a priori power calculation was performed due to limited prior data enabling reliable effect size estimation. Standardized effect sizes (Cohen’s d) were calculated for the primary outcomes and key comparisons in both study parts to enhance transparency.

Part A, an open-label, randomized, four-way crossover single-dose administration lasting for 60 min, randomized participants (using a computer-generated randomization list) into one of four treatments, a flavored nicotine pouch (ZYN), an unflavored nicotine pouch (ZYN), 10% sucrose (positive control), and 10% xylitol (negative control), with a one-week washout. Based on previous methodological experience with this plaque pH model, approximately 20 participants were considered sufficient to detect clinically relevant differences in plaque acidogenicity between the pouch products and the negative control.

Part B, an open-label, observational study focused on biofilm amount, acidogenicity, safety, and tolerability over a six-week period, had participants replace as many of their regular snus products as possible with ZYN products for the six-week study period. The frequency and timing of use were left to each participant. Anticipating a 25% dropout rate, the study aimed to include 60 participants to achieve 45 subjects.

### Product characteristics

All ZYN products (Swedish Match, Sweden) were stored in a refrigerator until use. In Part A, the following products were used to determine plaque acidogenicity: ZYN Dry Smooth (3 mg), ZYN Dry Peppermint (3 mg), 10% sucrose solution (positive control), and 10% xylitol solution (negative control).

In Part B, participants were given the option to select a 3- or 6-mg pouch of ZYN Dry Smooth, ZYN Dry Peppermint, or ZYN Dry Cinnamon throughout the ad libitum use for 6 weeks. Biofilm acidogenicity was assessed upon exposure to a 10% sucrose solution.

### Clinical variables

All tests and screenings were carried out at the research laboratory at the Department of Cariology, Institute of Odontology, University of Gothenburg. During screening for both study parts, individuals were asked about concomitant medication and baseline symptoms, and female participants underwent pregnancy testing. Tooth status – i.e., the sum of decayed, missing, and filled permanent teeth (DMFT) – gingival retraction, saliva secretion rate, and buffer capacity were evaluated via an oral examination.

In Part A, nicotine pouches were placed in the upper vestibule for 60 min during measurement to assess biofilm acidogenicity, while the positive and negative controls were administered as a mouth rinse for 60 s. The acidogenicity of dental biofilm was measured at baseline and at 2, 5, 10, 20, 30, 40, 50, and 60 min. The amount of biofilm was then evaluated. Participants returned weekly to take the tests.

Part B involved investigating eligible participants at baseline (week 0) and every two weeks (2, 4, and 6 weeks after product use). The following assessments were performed during each visit: oral cavity inspection, clinical photos, biofilm acidogenicity evaluation, salivary levels of oral microflora, and plaque amount. At each visit, the participants reported any local or general adverse events.

### Dental biofilm acidogenicity

Biofilm acidogenicity was measured using the microtouch method [16]. An iridium microelectrode (Beetrode^®^ NEPH, WPI Instruments, New Haven, Conn., USA) was inserted into the biofilm on two approximal surfaces in the premolar/molar region. The measuring electrode and a reference electrode (Dri-Ref™ 5SH, WPI Instruments, New Haven, Conn., USA) were connected to an Orion SA720 pH/ISE Meter (Orion Research, Boston, Mass., USA). The reference electrode and the participant’s finger were submerged in a 3-M KCl solution to create a salt bridge while measuring pH. Before and during each test, the electrode was calibrated against a standard buffer at pH 7.00 (Merck KGaA, Darmstadt, Germany). The area under the curve (AUC_7.5_) for dental plaque acidogenicity was calculated as the area between the reference line (pH 7.5) and the recorded plaque pH curve over the 60-minute measurement period. The AUC was computed using the trapezoidal method based on serial pH measurements obtained at predefined time intervals.

### Plaque amount

The plaque amount was assessed by the index described by Silness and Löe (1964) [17]. Plaque quantity was scored on six teeth (mesio-buccal, buccal, disto-buccal, disto-lingual, lingual, and mesio-lingual) and rated (0–3) for each surface.

### Saliva sampling

Unstimulated saliva was obtained by drooling for 5 min. The stimulated saliva was collected by chewing on a piece of paraffin and continuously spitting saliva into a graded test tube for 5 min. Secretion rates were calculated in mL/min. One mL of collected saliva was transferred to Viability-preserving Microbistatic Medium II for microbiological analysis, and 1 mL was used for buffer capacity. Buffer capacity was assessed using the technique described by Ericsson (1959) and determined as final pH [18].

### Oral microflora

Saliva samples were dispersed using a Whirlimixer and serially diluted (10-fold) in sterile potassium phosphate buffer. Aliquots (25 µL) from appropriate dilutions were plated in duplicate on Mitis Salivarius agar with bacitracin (MSB) to estimate *S. mutans* level and Rogosa agar to estimate total lactobacilli count. After incubation in its respective atmosphere for 48–72 h at 37 °C, colony-forming units (CFU) were counted. The culture media were prepared at the department according to standard laboratory procedures. All plates were incubated at 37 °C in a controlled atmosphere of 90% N₂ and 10% CO₂ for approximately 4 days.

After incubation, colony-forming units (CFU) were counted. Plates containing countable numbers of colonies were included in the analysis. Bacterial concentrations were expressed as CFU/mL, taking the dilution factor into account. The mean value of duplicate plates was used.

### Statistical analysis

Continuous variables are presented as number (N), mean, median, and standard deviation (SD). For plaque pH, baseline pH, minimum pH, final pH, and the area under the curve below pH 7.5 (AUC_7.5_) were calculated. Standardized effect sizes were calculated as Cohen’s d using the difference in means divided by the pooled standard deviation.

Given the within-subject nature of the study components, repeated-measures analytical approaches were applied. When more than two related conditions were compared, one-way repeated measures ANOVA was used. When the overall model was significant, post hoc multiple comparisons were performed using Tukey’s test. For comparisons between two related time points, paired analyses were conducted using the Wilcoxon matched-pairs signed-rank test where appropriate.

All statistical analyses were performed using GraphPad Prism (GraphPad Software Inc., San Diego, CA, USA). A two-sided p-value < 0.05 was considered statistically significant.

## Results

### Number of participants

A total of 83 participants (23 participants in Part A and 60 participants in Part B) were screened. In total, 79 participants fulfilled all inclusion criteria. Screening failure accounted for the non-inclusion of three participants in Part A and one participant in Part B. Table 1 lists the baseline characteristics of the participants. Of the 20 participants enrolled in Part A, 18 completed the study. The two participants who withdrew cited personal reasons for their withdrawal. Of the 59 participants enrolled in Part B, 56 completed the study, two withdrew for personal reasons, and one was excluded as the use of hypertension medication became evident.

**Table 1:** Baseline and demographic characteristics of participants

		**PART A (n=20)**	**PART B (n=59)**
Age (years)	Mean (SD)	34.6 (11.1)	31.4 (10.1)
	Median (Min, Max)	30.5 (23, 64)	27 (21, 64)
Gender	Female	6 (30%)	20 (34%)
	Male	14 (70%)	39 (66%)
Saliva Buffer Capacity (pH)	Mean (SD)	6.27 (1.38)	6.18 (1.25)
	Median (Min, Max)	6.55 (3.6, 8.0)	6.4 (3.6, 8.0)
Stimulated Saliva Secretion Rate (mL/min)	Mean (SD)	2.28 (1.53)	2.32 (1.04)
	Median (Min, Max)	1.95 (1.1, 8.0)	2.1 (0.7, 5.0)
Unstimulated Saliva Secretion Rate (mL/min)	Mean (SD)	0.315 (0.22)	0.51 (0.37)
	Median (Min, Max)	0.2 (0.1, 1.0)	0.4 (0.1, 1.7)
Tooth Status (DMFT)	Mean (SD)	6.1 (5.87)	3.7 (4.60)
	Median (Min, Max)	4.5 (0, 20)	2.0 (0, 20)

Summary of demographic data and other baseline characteristics of the participants in Part A and Part B

### Adverse events

The administration of ZYN proved to be safe and well-tolerated by the healthy participants. No serious adverse events or discontinuations attributed to adverse events were observed throughout the study. The five adverse events related to the study products included two cases of mild nausea, one case of mild dry mouth, one case of moderate gingival blisters, and one case of mild dizziness.

### Part A

#### Dental plaque acidogenicity after short-term exposure

**Fig. 1:**
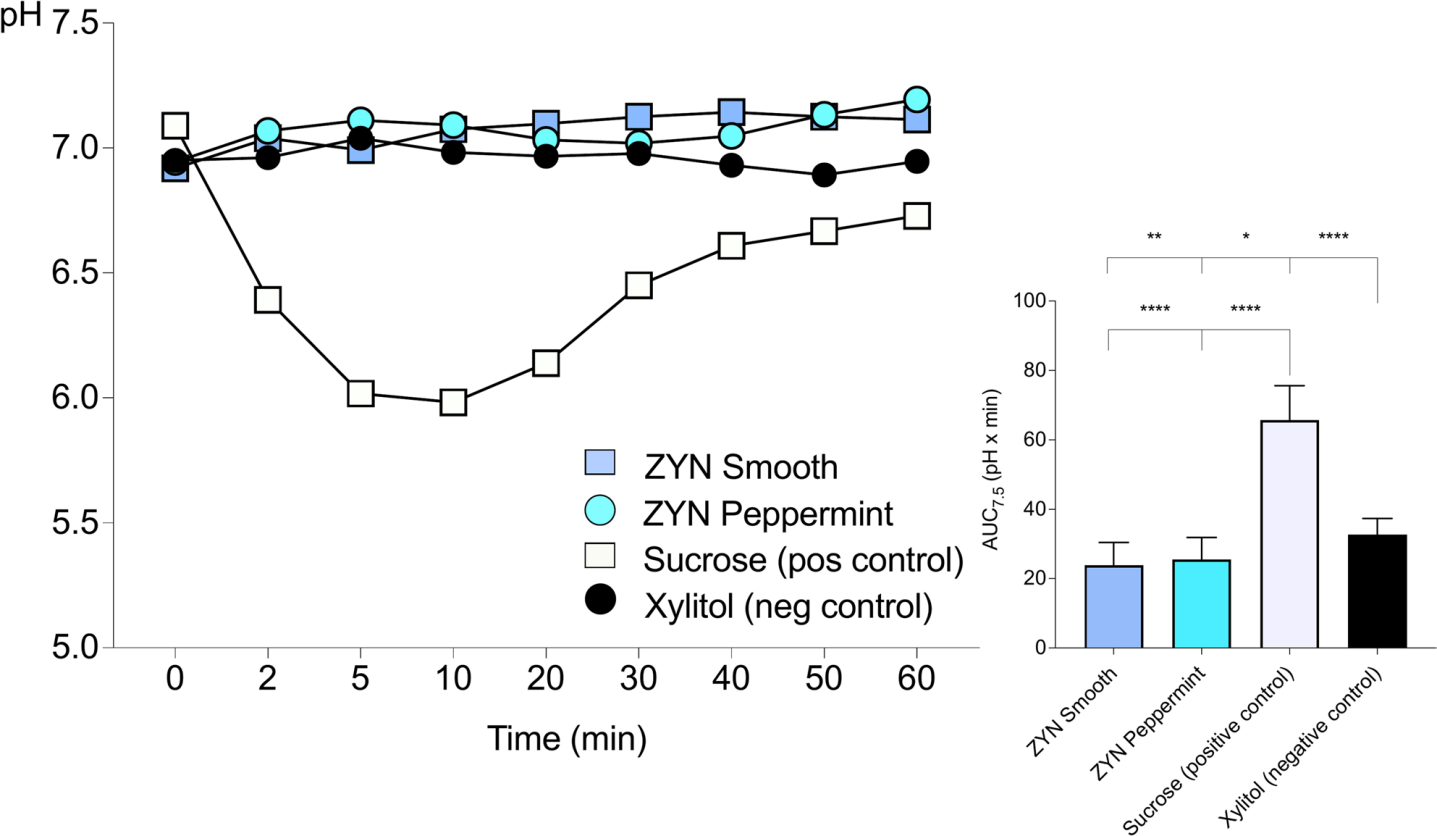
Dental plaque acidogenicity after single-dose administration (Part A). Mean pH values in dental plaque over 60 min following the use of ZYN Smooth, ZYN Peppermint, 10% sucrose rinse, and 10% xylitol rinse. The area under the curve below pH 7.5 (AUC_7.5_) reflects plaque acidogenicity over time. Statistical analysis was performed using one-way ANOVA. ^****^p < 0.0001, ^**^p < 0.01, ^*^p < 0.05. *n* = 18

The results for plaque pH acidogenicity in Part A are visualized in Fig. 1, with the corresponding descriptive statistics presented as mean values for the two sites in Table 2. A clear negative curve was observed after the sucrose rinse (positive control), while an increase in plaque acidogenicity was noted for the two ZYN products and after the xylitol (negative control) rinse. Four statistically significant differences in the AUC_7.5_ were identified: between both ZYN products and sucrose (*p* < 0.0001); between xylitol and sucrose (*p* < 0.0001); between xylitol and ZYN Smooth (*p* < 0.05); and between xylitol and ZYN Peppermint (*p* < 0.01). The standardized effect sizes for the comparisons between the nicotine pouch products and xylitol were large (ZYN Smooth vs. xylitol: Cohen’s d = 1.54; ZYN Peppermint vs. xylitol: Cohen’s d = 1.29).

Statistical analysis was conducted to assess differences in pH values measured at baseline, final pH, and the minimum pH levels measured after using ZYN products and rinsing with the controls. There were no statistically significant differences in baseline pH values. However, statistically significant differences were found in the minimum pH values measured after using ZYN Smooth, ZYN Peppermint, and xylitol rinse (*p* < 0.0001) compared to the sucrose rinse. Additionally, statistically significant differences were observed in the final pH measured after using ZYN Smooth (*p* < 0.05) and ZYN Peppermint (*p* < 0.01) compared to the sucrose rinse.

**Table 2:**
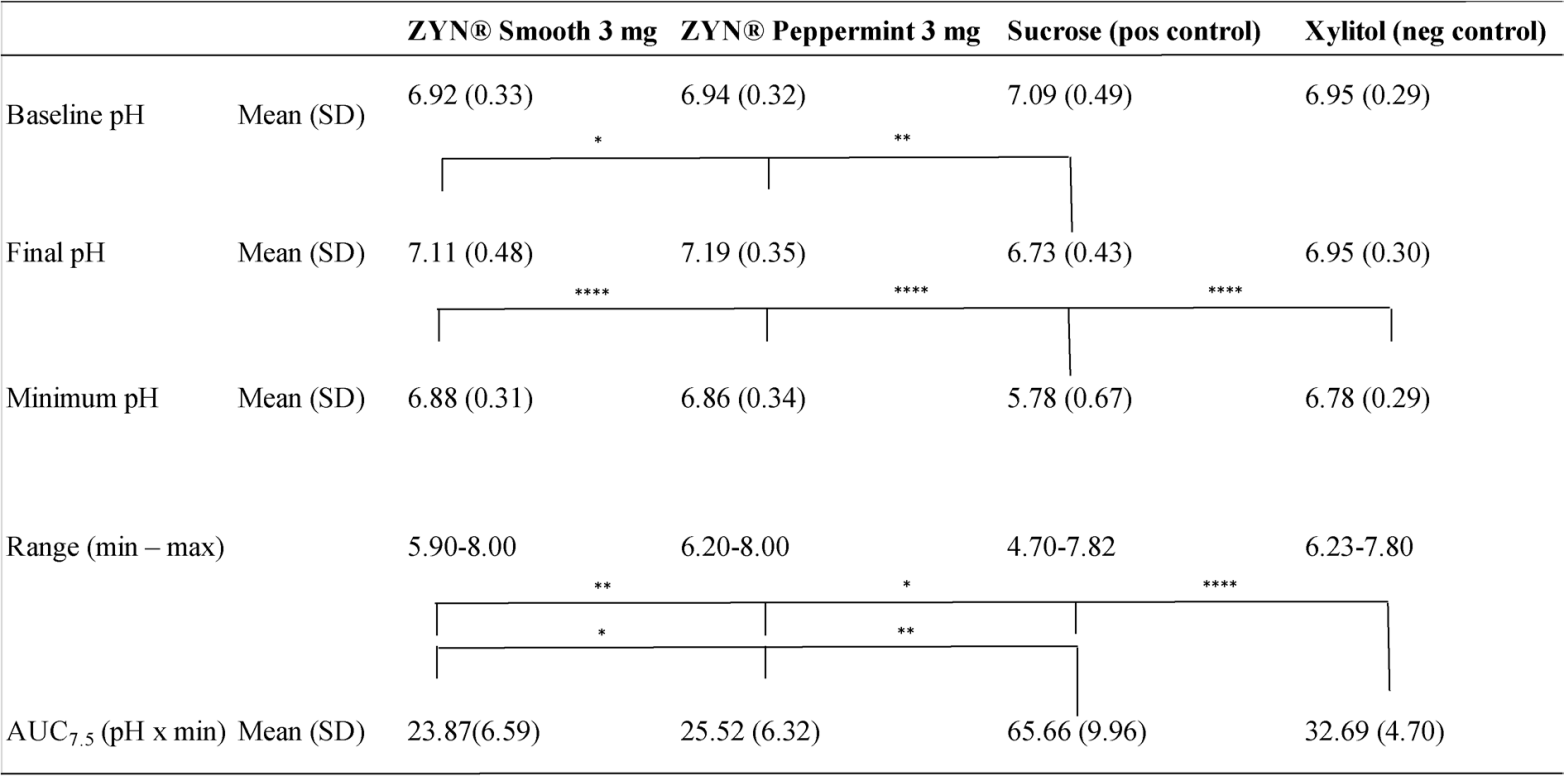
Dental plaque acidogenicity Part A

Dental plaque acidogenicity from Part A presented as baseline pH, minimum pH, final pH, range (min–max) as well as AUC_7.5_ (mean ± SD) for the mean of the two different sites

Statistical analysis was performed using one-way ANOVA; ^****^p < 0.0001, ^**^p < 0.01, ^*^p < 0.05. *n* = 18

### Part B

#### Treatment compliance

Between visits, participants were allowed to use multiple nicotine-containing products (including tobacco-based products). However, they were encouraged to replace as many of these products with ZYN (ZYN Peppermint, Cinnamon, and Smooth, each containing 3 or 6 mg of nicotine) as possible. Throughout the study, the proportion of ZYN product use increased in relation to the total number of nicotine products used. The mean and SD ratios (ZYN/total number of nicotine-containing products used) were 0.72 (0.22) from baseline to week 2, 0.81 (0.18) from week 2 to week 4, and 0.84 (0.16) from week 4 to week 6.

#### Dental plaque acidogenicity after 6 weeks of exposure

**Fig. 2:**
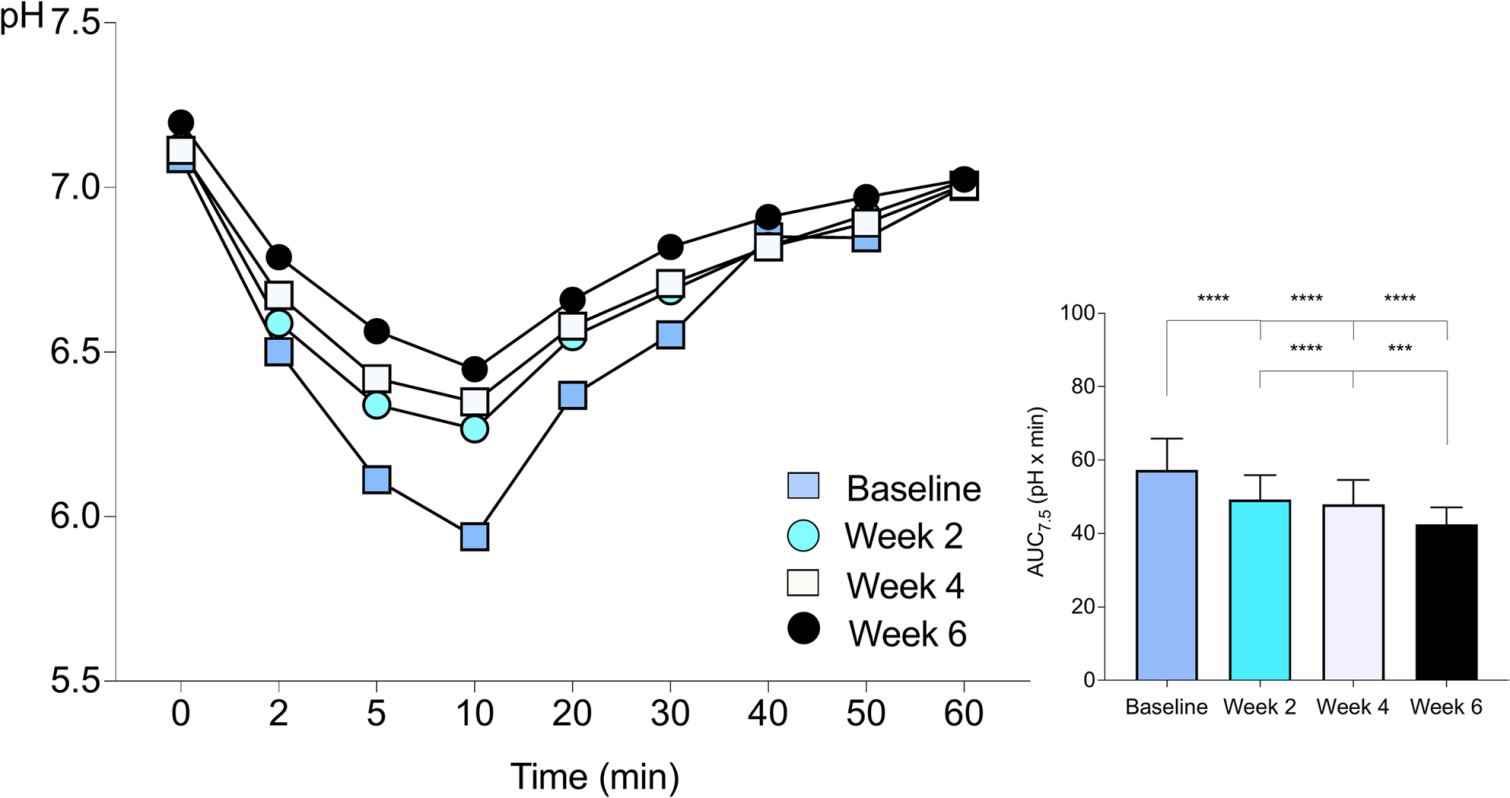
Dental plaque acidogenicity after ad libitum administration (Part B). Mean pH values in dental plaque before and after a 10% sucrose rinse across four visits (baseline (week 0), 2, 4, and 6 weeks). The area under the curve below pH 7.5 (AUC_7.5_) represents plaque acidogenicity over time. Statistical analysis was performed using one-way ANOVA. ^****^p < 0.0001, ^***^p < 0.001. *n* = 56

Figure 2 presents data on plaque pH acidogenicity for Part B after the sucrose rinse. Table 3 lists the corresponding descriptive statistics as mean values for the two sites. The most attenuated pH fall was observed at baseline (week 0), followed by a gradual increase in pH from week 2 to week 4 and from week 4 to week 6. This was accompanied by a corresponding decrease in the AUC_7.5_. Statistically significant differences were found when comparing AUC_7.5_ between all the different visits (*p* < 0.001 or *p* < 0.0001), except for week 2 compared to week 4. The change from baseline to week 6 demonstrated a large, standardized effect size (Cohen’s d = 2.18).

Statistical analysis was conducted to assess differences in pH values measured at baseline (before sucrose rinse), final pH, and minimum pH levels at baseline (week 0), week 2, week 4, and week 6. No statistically significant differences were found in baseline pH values between the different time points. However, significant differences were found in the minimum pH values after 2 weeks (*p* < 0.01), 4 weeks (*p* < 0.001), and 6 weeks (*p* < 0.0001) of using ZYN products compared to baseline (week 0). Additionally, statistically significant differences were observed in the final pH after 6 weeks compared to 4 weeks (*p* < 0.01), 2 weeks (*p* < 0.01), and baseline (week 0) (*p* < 0.01).

**Table 3:**
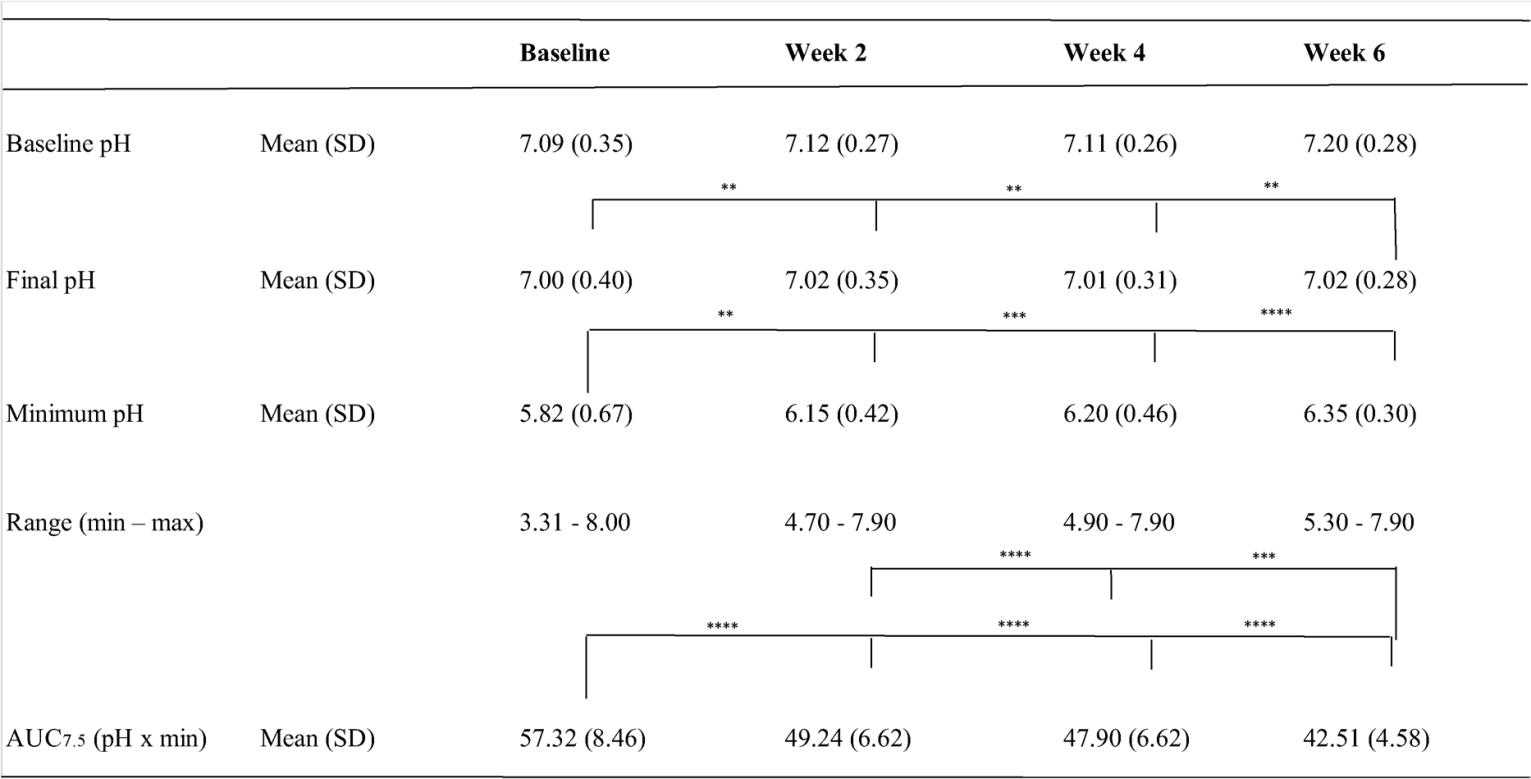
Dental plaque acidogenicity Part B

Dental plaque acidogenicity from Part B presented as baseline pH, minimum pH, final pH, range (min–max) as well as AUC_7.5_ (mean ± SD) for the mean measured at the two different sites in snus users who substituted their regular snus as much as possible with ZYN products (Part B)

Statistical analysis was performed using one-way ANOVA; ^****^p < 0.0001, ^***^p < 0.001, ^**^p < 0.01. *n* = 56

#### Plaque amount

**Fig. 3:**
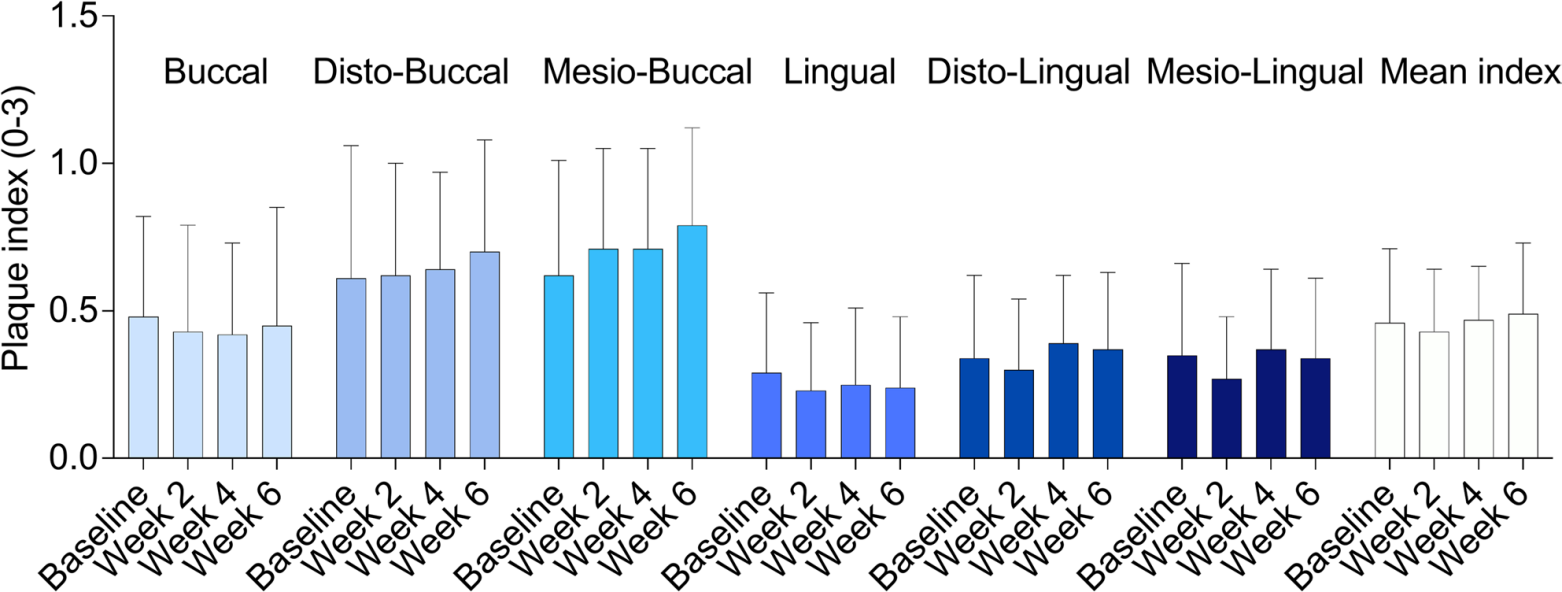
Plaque levels across different tooth surfaces (Part B). The amount of plaque scored from 0 to 3 is presented for the six tooth surfaces: mesio-buccal, buccal, disto-buccal, disto-lingual, lingual, mesio-lingual, and as a mean index. Data are presented as mean ± SD. *n* = 56

The mean plaque index for the six tooth sites varied: 0.46 ± 0.25 (baseline), 0.43 ± 0.21 (week 2), 0.47 ± 0.18 (week 4), and 0.49 ± 0.24 (week 6) (Fig. 3). No statistically significant changes in mean plaque index were found throughout the six weeks for the mean or any of the individual sites. Additionally, plaque amounts were numerically higher throughout the entire test period for the buccal sites compared to the lingual sites.

#### Oral microflora

**Fig. 4:**
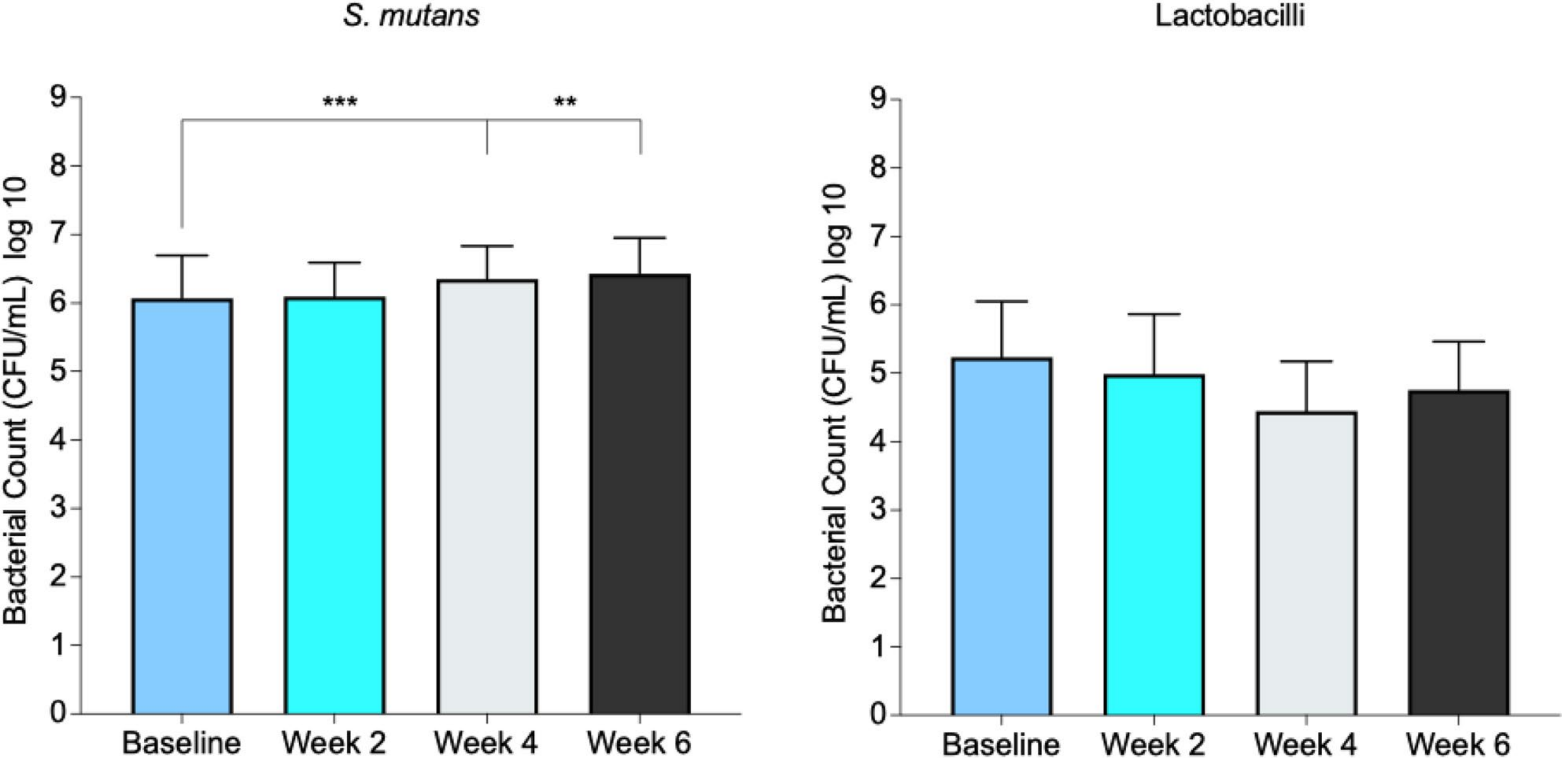
Salivary growth of *Streptococcus mutans* (*S. mutans*) and lactobacilli before and after substitution of regular snus with ZYN products (Part B). Bacterial growth was assessed at four visits (baseline (week 0), 2, 4, and 6 weeks). Data are presented as log CFU/mL (median ± interquartile range). Statistical analysis was performed using the Wilcoxon matched-pairs signed-rank test. ^***^p < 0.001, ^**^p < 0.01. *n* = 56

A statistically significant increase (*p* < 0.05) in *S. mutans* levels was observed at weeks 4 and week 6 compared to baseline. Although no statistically significant changes were found for lactobacilli throughout the study, a numerical decrease was observed from baseline to week 6 (Fig. 4).

## Discussion

The primary objective of this study was to evaluate the safety and tolerability of ZYN, a nicotine pouch product devoid of tobacco leaf, with a particular focus on its potential to impact biofilm acidogenicity. In Part A, dental plaque acidogenicity was assessed following short-term exposure (60 min) to both flavored and unflavored ZYN variants. Simultaneously, positive (10% sucrose rinse) and negative (10% xylitol rinse) controls were assessed. Following short-term exposure, the AUC_7.5_ for dental plaque acidogenicity was significantly lower for ZYN products and xylitol compared to sucrose. Part B aimed to assess dental plaque acidogenicity over a six-week period of ad libitum ZYN use. Although the number of lactobacilli remained unchanged, the number of *S. mutans* statistically significantly increased after using ZYN. Despite this increase, after six weeks of using ZYN, there was a less pronounced dental plaque acidogenicity following a sucrose rinse, suggesting a possible reduction in the cariogenic potential of the oral microflora. Furthermore, this study was part of a larger investigation primarily focused on snus-induced lesions (SILs). ZYN products also had a positive effect on these lesions, leading to improvement of pre-existing SILs over the course of the study compared to baseline [19].

Swedish snus, distinct from other forms of moist snuff, is a type of oral tobacco that adheres to the GothiaTek standard, meaning it contains significantly lower levels of carcinogenic nitrosamines [20, 21]. These products are widely used in Sweden, Norway, Switzerland, and the U.S [22, 23]. Swedish snus is composed of finely ground dried tobacco, water, moisturizers, sodium chloride (NaCl), sodium carbonate, and flavorings [23]. The product is typically placed in the sulcus under the upper or lower lip, allowing direct contact with the oral mucosa. This placement leads to efficient nicotine absorption through the mucosal membrane into the bloodstream, resulting in significant nicotine intake [24]. The pH of Swedish snus is relatively high (pH 8.7) compared to other tobacco products such as American snus (pH 6.5) and cigarettes [25, 26]. It has been suggested that the high pH of Swedish snus may increase the pH in the oral cavity. This elevated pH could promote the remineralization of tooth surfaces and inhibit cariogenic, acid-producing microflora [7].

This study evaluates the safety and tolerability of the nicotine pouch product ZYN, with a particular focus on its potential to impact biofilm acidogenicity and, consequently, caries disease. ZYN pouch products contain approximately 3% moisture. Each pouch (14 × 28 mm; 0.4 g) contains nicotine in salt form along with fillers (maltitol and microcrystalline cellulose), a stabilizer (hydroxypropyl cellulose), pH adjusters (sodium carbonate and sodium bicarbonate), food-grade flavorings, and the sweetener acesulfame K. The nicotine content varies depending on product strength. Previous studies have shown that bacteria such as *S. mutans* and lactobacilli are closely linked to the development of dental caries and are among the primary agents of this disease [27–29]. Both *S. mutans* and lactobacilli are rapid acid producers from simple carbohydrates, potentially increasing the risk of cavities by creating an acidic environment [29]. Hellqvist et al. reported no significant differences in the salivary counts of *S. mutans* or lactobacilli between Swedish snus users and controls [1]. However, other studies have suggested that the nicotine found in smokeless tobacco could promote the growth of *S. mutans* [4, 5]. In the current study, there was no statistically significant increase in plaque amount or change in the number of lactobacilli between baseline and subsequent visits. However, the number of *S. mutans* significantly increased after 4 and 6 weeks of using ZYN products compared to baseline (week 0).

Concerning plaque acidogenicity, previous studies have reported no differences in dental plaque pH between Swedish snus users and controls; however, these studies have found a tendency towards a more pronounced decrease in pH among non-snus users compared to snus users [1]. Similar findings have been reported for snus products, which were found to increase pH levels in plaque [2]. In Part A of the present study, the AUC for dental plaque acidogenicity did not show any statistically significant differences between the ZYN products and xylitol (negative control). In fact, the minimum pH and the final pH values observed during the measurement were numerically more favorable after administration of ZYN products compared to xylitol. After ad libitum administration of ZYN in Part B, the results showed decreasing AUC values when calculated below pH 7.5 over time for dental plaque acidogenicity. This reduction in AUC suggests reduced acid production by dental plaque over time, which may help lower the risk of developing dental caries. Additionally, the minimum pH values observed at weeks 2, 4, and 6 showed a statistically significant increase compared to baseline (week 0). Furthermore, the final pH values measured at week 6 were significantly higher compared to those at week 2 and baseline. The observed improvement in plaque pH after 6 weeks of using ZYN products indicates a decrease in acidity within the dental plaque. This reduction in acidogenicity may be beneficial as it lowers the risk of demineralization of tooth enamel, a precursor to caries lesions.

The number of *S. mutans* and lactobacilli, plaque pH measurements, and plaque amount were assessed as surrogate markers related to cariogenic biofilm activity. As previously noted, there was no statistically significant change in the number of lactobacilli; however, the number of *S. mutans* was significantly higher after the use of ZYN products compared to baseline levels. Interestingly, in Part B, despite an increase in the population of the acid-producing bacteria *S. mutans*, the use of ZYN for 6 weeks resulted in less pronounced biofilm acidogenicity. This indicates that changes in plaque pH dynamics were not directly proportional to the observed increase in this bacterial species. The change in dental plaque pH may, aside from *S. mutans* and lactobacilli, also have resulted from a reduction in other acid-producing bacteria and/or their characteristics following the use of ZYN. This altered environment may have been more beneficial to certain bacteria, such as *S. mutans*, while being less suitable for others, which could explain the observed findings. However, it is important to emphasize that plaque acidogenicity represents a surrogate parameter, and no clinical caries outcomes were assessed in the present study. Nonetheless, it remains possible that other factors, such as changes in diet or oral hygiene practices also played a role in shaping the composition of the oral microbiota over time.

The main limitation of the present study is that, despite encouraging participants to substitute their previous snus products with ZYN as much as possible (ideally all), they were still allowed to combine their old snus with ZYN. It would have been ideal for participants to completely abandon their snus products upon starting the use of ZYN. A valuable consideration for future studies would be to implement a restriction that allows participants to use only the new products, prohibiting the use of regular snus. Additionally, it would be worthwhile to investigate a broader range of cariogenic bacteria in future research.

Finally, although no control group was included in Part B, outcomes were compared within the same individuals before and after switching, with baseline serving as the reference condition. This within-subject design reduces inter-individual variability. However, the absence of a parallel control group may limit the ability to account for potential time-related confounding.

## Conclusion

Within the limitations of this exploratory study, single-dose administration of a non-tobacco nicotine pouch was associated with a decrease in dental plaque acidogenicity in healthy snus users. Following 6 weeks of ad libitum use, a less pronounced biofilm acidogenicity was observed compared with baseline.

These findings reflect changes in surrogate biofilm parameters within the time frame studied and should not be interpreted as evidence of clinical caries outcomes. Further studies with extended follow-up and clinical endpoints are warranted to better understand the long-term implications.

## Data Availability

The datasets used and/or analysed during the current study are available from the corresponding author on reasonable request.
